# MSSFNet: A Multiscale Spatial–Spectral Fusion Network for Extracting Offshore Floating Raft Aquaculture Areas in Multispectral Remote Sensing Images

**DOI:** 10.3390/s24165220

**Published:** 2024-08-12

**Authors:** Haomiao Yu, Yingzi Hou, Fangxiong Wang, Junfu Wang, Jianfeng Zhu, Jianke Guo

**Affiliations:** 1 School of Geographical Sciences, Liaoning Normal University, Dalian 116029, China; yhmhhxx@dlmu.edu.cn (H.Y.); geoffrey@lnnu.edu.cn (J.W.); zjf014@lnnu.edu.cn (J.Z.); 2 Liaoning Provincial Key Laboratory of Physical Geography and Geomatics, Liaoning Normal University, Dalian 116029, China; 3 Institute of Marine Sustainable Development, Liaoning Normal University, Dalian 116029, China

**Keywords:** floating raft aquaculture, multispectral remote sensing images, sentinel-2 data, spectral and spatial information, deep learning

## Abstract

Accurately extracting large-scale offshore floating raft aquaculture (FRA) areas is crucial for supporting scientific planning and precise aquaculture management. While remote sensing technology offers advantages such as wide coverage, rapid imaging, and multispectral capabilities for FRA monitoring, the current methods face challenges in terms of establishing spatial–spectral correlations and extracting multiscale features, thereby limiting their accuracy. To address these issues, we propose an innovative multiscale spatial–spectral fusion network (MSSFNet) designed specifically for extracting offshore FRA areas from multispectral remote sensing imagery. MSSFNet effectively integrates spectral and spatial information through a spatial–spectral feature extraction block (SSFEB), significantly enhancing the accuracy of FRA area identification. Additionally, a multiscale spatial attention block (MSAB) captures contextual information across different scales, improving the ability to detect FRA areas of varying sizes and shapes while minimizing edge artifacts. We created the CHN-YE7-FRA dataset using Sentinel-2 multispectral remote sensing imagery and conducted extensive evaluations. The results showed that MSSFNet achieved impressive metrics: an F1 score of 90.76%, an intersection over union (IoU) of 83.08%, and a kappa coefficient of 89.75%, surpassing those of state-of-the-art methods. The ablation results confirmed that the SSFEB and MSAB modules effectively enhanced the FRA extraction accuracy. Furthermore, the successful practical applications of MSSFNet validated its generalizability and robustness across diverse marine environments. These findings highlight the performance of MSSFNet in both experimental and real-world scenarios, providing reliable, precise FRA area monitoring. This capability provides crucial data for scientific planning and environmental protection purposes in coastal aquaculture zones.

## 1. Introduction

The ocean environment is a major source of high-quality protein for humans, and modernized ocean ranching is a new model for the sustainable development of fisheries [1]. In recent years, the marine ranching and aquaculture industry of China has developed rapidly, which has brought considerable economic benefits to society but also presents great challenges to marine hydrodynamics and the ecological environment [2]. The uncontrolled expansion of marine pastures and increasing aquaculture densities may jeopardize the sustainable development of marine ecosystems and the safety of maritime transportation and biodiversity [3]. Marine floating raft aquaculture (FRA) is an important component of marine pastures and is the most commonly used coastal aquaculture method. Therefore, rapidly and accurately monitoring the distribution and quantity of FRA regions helps facilitate scientific aquaculture management and planning and is highly important for promoting green offshore ecological development.

The traditional FRA monitoring methods, including field surveys and statistical analyses, not only involve high labor intensity levels and costs but are also susceptible to weather conditions and human errors. In contrast, remote sensing technology, with its advantages such as large-area coverage, rapid imaging, multispectral bands, and cost-effectiveness, has become an effective tool for the large-scale monitoring and extraction of nearshore aquaculture regions [4].

To improve the accuracy and efficiency of extracting FRA areas from remote sensing images (RSIs), researchers have proposed many related methods that incorporate tools such as spectral feature extraction [5,6], object-oriented extraction [7,8,9], and machine learning algorithms (decision trees, support vector machines, random forests, etc.) [10,11,12] into FRA recognition. However, methods based on spectral characteristics face the problem that different objects may have similar spectral characteristics, which can easily lead to confusion. The thresholds set in object-oriented methods are generally aimed only at specific research fields and, consequently, the portability and generalizability of these methods are poor. Moreover, traditional machine learning methods exhibit poor robustness when applied to challenging water information-related tasks and cannot extract targets from complex images, resulting in low extraction accuracy.

In recent years, deep learning, especially semantic segmentation technology, has demonstrated excellent feature extraction capabilities in FRA areas monitoring tasks. Compared with other methods, semantic segmentation provides higher extraction accuracy and stronger generalizability. For example, Chen et al. combined DeepLabv3+ and UNet with Gaofen (GF)-1 RSIs to apply them to the large-scale extraction of marine aquaculture areas, demonstrating that semantic segmentation models have superior recognition and generalization capabilities [13]. Similarly, Ai et al. integrated GF-2 RSIs with a self-attention mechanism and an auxiliary loss network to achieve high-precision aquaculture area extraction, providing data support for the rational planning and environmental protection of coastal aquaculture zones [14]. Cheng et al. designed HDCUNet by combining UNet with hybrid dilated convolution (HDC) to expand the receptive field of their network, which was used to extract coastal aquaculture areas from GF-2 RSIs, achieving significantly improved extraction accuracy [15]. Despite these significant advancements, existing methods based on high-resolution visible-light imagery often struggle when addressing backgrounds that have similar spectral characteristics, such as suspended sediment or water surface shadows. The limited number of available spectral bands can result in the loss of feature information, affecting the achieved extraction accuracy. Furthermore, high acquisition costs and long satellite revisitation cycles limit the economic feasibility and timeliness of nearshore aquaculture monitoring.

To address these limitations, researchers have explored the potential of multispectral RSIs. Although multispectral imagery sacrifices some spatial resolution, it covers multiple spectral bands from the visible to the near-infrared region, providing a broader range of spectral information than the visible spectrum alone does [16,17]. This richness of information is particularly useful in area extraction and large-scale monitoring tasks in complex environments, compensating for the spectral limitations of high-resolution imagery. Additionally, multispectral imagery is relatively easy to obtain, has a low cost, and has shorter satellite revisit periods, further enhancing its suitability for large-scale, frequent monitoring tasks. For example, Su et al. proposed RaftNet based on UNet to accurately extract FRA regions from Landsat 8 operational land imager (OLI) images, and achieved high FRA extraction accuracy in Sansha Bay, Fujian Province [18]. Lu et al. combined Sentinel-2 multispectral image data and an improved UNet to identify aquaculture areas accurately, significantly reducing the degree of edge adhesion in aquaculture areas [19]. Liu et al. integrated Sentinel-2 multispectral imagery with spectral indices and designed the SRUNet model to accurately extract nearshore raft aquaculture areas from medium-resolution remote sensing imagery [20].

However, existing methods typically only use data acquired from different spectral ranges of medium-resolution multispectral imagery as inputs for their models, extracting their spatial features through convolutional neural networks (CNNs). This approach often overlooks a crucial dimension of multispectral imagery: its spectral features. In fact, these spectral bands contain rich and complementary feature information, which is important for improving the accuracy of offshore aquaculture zone extraction. Many studies have integrated the channel attention (CA) mechanism into remote sensing image processing models to calculate the importance of each spectral channel [21,22]. However, this approach does not establish relationships between multiple channels and, thus, fails to promote information interaction between different spectral features, resulting in the failure to fully explore and utilize the intrinsic connections and complementary information between spectral bands. Therefore, how to effectively integrate and utilize multispectral and spatial information remains a key challenge for improving the efficiency and accuracy of FRA extraction.

In addition, the scale variations exhibited by remotely sensed imagery cause target features to appear in different areas of images as different sizes. The scales and spatial extents of these features in remotely sensed imagery are broader and more complex than those in conventional imagery, which makes it difficult for single-scale feature extraction to fully capture these variations [23,24]. To address this challenge, researchers have integrated multiscale feature extraction methods, such as dilated convolution (DC) [25,26] or Vision Transformers (ViTs) [27], into their models to increase their feature extraction capabilities in FRA regions of different sizes, thus significantly improving the resulting extraction accuracy [15,20]. Although the DC method extends the receptive field of a CNN and enhances its ability to capture multiscale features to a certain extent, it still relies on the local receptive field and is unable to effectively capture long-distance information from large-scale RSIs, which leads to performance limitations [28,29]. The advantages of ViTs are their global self-attention mechanisms and flexible structures, which enable them to perform well on large datasets and handle long-distance dependencies. However, ViTs have high computational and memory overheads, high data volume requirements, and difficult training optimization processes. In practical applications, it is often necessary to balance these factors and possibly incorporate other methods (e.g., CNNs) to compensate for the shortcomings of ViTs [30,31]. Therefore, establishing effective multiscale or global contextual relationships in a model is crucial for improving its performance.

To address the insufficient utilization of spectral features and the limitations of single-scale spatial feature extraction in offshore aquaculture area extraction based on multispectral RSIs in the above studies, we propose a new multiscale spatial–spectral fusion network (MSSFNet). This network aims to improve the accuracy of offshore aquaculture area extraction in multispectral RSIs. The contributions of this paper are as follows:

- To improve the accuracy of FRA region extraction in multispectral images, we designed a new semantic segmentation model, MSSFNet, on the basis of an encoder–decoder structure. In the encoder part of the network, we develop a spatial–spectral feature extraction block (SSFEB), which improves the defect concerning the underutilization of spectral information in the traditional convolutional method and efficiently fuses spectral features and spatial information to improve the accuracy of FRA region recognition.
- In MSSFNet, we designed a multiscale spatial attention block (MSAB). This block implements a global receptive field and multiscale feature learning, which enhances the adaptability of the network to complex backgrounds, makes the FRA region extraction process more accurate and robust, and improves the ability of the model to identify and segment the target region in complex RSIs.
- We construct the CHN-YE7-FRA dataset for FRA extraction on the basis of Sentinel-2 multispectral remote sensing images, which solves the current problem of missing sample data for offshore FRA area extraction. The dataset accounts for the differences in depth, color, and shape of aquaculture areas in different Chinese seas, annotates representative FRA areas in multiple seas, enhances intraclass diversity, and provides important support for future FRA extraction studies under different environments and conditions.

## 2. Materials

### 2.1. Study Areas

**Figure 1:**
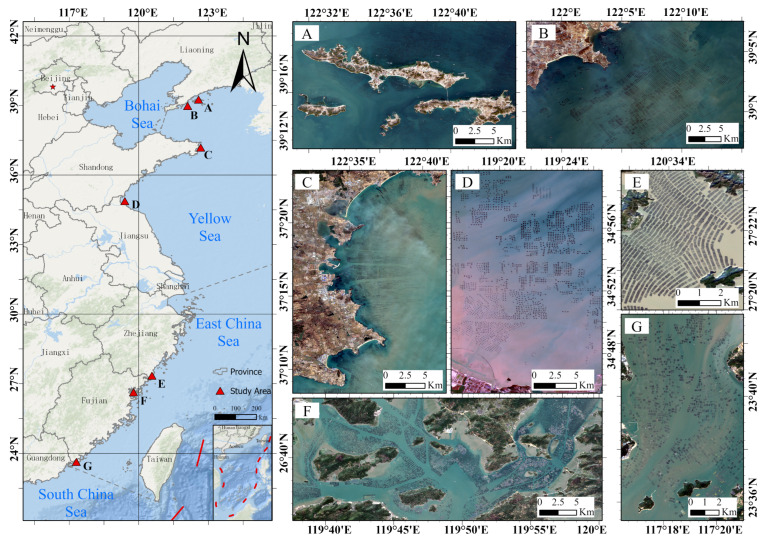
Geographical locations of and images derived from the study areas ((**A**). Changhai County in Liaoning Province, (**B**). Jinshitan Bay in Liaoning Province, (**C**). Rongcheng Bay in Shandong Province, (**D**). Haizhou Bay in Jiangsu Province, (**E**). Dayu Bay in Jiangsu Province, (**F**). Sansha Bay in Fujian Province, and (**G**). Zhaoan Bay in Fujian Province).

China has a unique geographical location and a vast coastline, and its rich marine resources support various forms of marine economic activities. Mariculture in coastal areas is an important part of the national economy of China, and various types of aquaculture areas are widely distributed along coastlines from north to south, forming a unique offshore aquaculture landscape [12]. Offshore floating raft aquaculture, as an important aquaculture method, is particularly common in offshore areas of China. This method uses floating rafts and ropes to form culture racks (which are anchored through the seabed for the cultivation of marine organisms) and is particularly suitable for the propagation of seaweeds and attached animals (e.g., shellfish and oysters). These organisms are attached to ropes suspended below floating rafts, creating a low-cost, short-cycle, and efficient culture method [32]. In this study, we focused on seven typical offshore FRA areas in the Yellow Sea and East China Sea as sample collection sites, including Changhai County, Liaoning Province, Jinshitan Bay, Liaoning Province, Rongcheng Bay, Shandong Province, Haizhou Bay, Jiangsu Province, Dayu Bay, Zhejiang Province, Sansha Bay, Fujian Province, and the vicinity of Zhaoan Bay, Fujian Province. Each of these areas has its own characteristics, but they generally have moderate water depths, slow current speeds, and fertile water quality levels, which are very suitable for offshore FRA [33,34,35,36,37,38]. The regions selected in this paper not only fully represent the differences in mariculture between northern and southern China but also cover multiple latitudes and climate zones with diverse geological conditions. This diversity ensures the comprehensiveness and representativeness of the samples, which can largely improve the generalizability of the model and make it suitable for multi-region FRA extraction work. In an RSI, the reflectivity of FRA is generally lower than that of the surrounding seawater, so this type of aquaculture area usually appears with a regular black and gray strip distribution in the image. The geographical distribution of each area and the typical characteristics of FRA areas are shown in Figure 1.

### 2.2. Dataset and Data Processing

To address the lack of sample data in offshore floating raft aquaculture areas, a new dataset named CHN-YE7-FRA was developed. This dataset aims to support research on offshore FRA area extraction. This dataset was constructed via Sentinel-2 multispectral remote sensing imagery obtained from the European Space Agency (ESA) (https://www.esa.int/, accessed on 11 March 2024). These images, acquired by a multispectral instrument (MSI), cover 13 spectral bands ranging from visible to near-infrared, with a spatial resolution of 10 m and a revisitation period as short as 5 days [39]. To increase the diversity and representativeness of the dataset, we selected images from December 2022 and January 2023, which had lower degrees of cloud cover and reduced spectral reflectance in the FRA areas. During these months, the spectral reflectance difference between the FRA areas and the background seawater is minimal, which helps mitigate the impacts of seasonal spectral variations on the accuracy of FRA extraction. Using the Google Earth Engine (GEE) (https://developers.google.com/earth-engine/, accessed on 11 March 2024) platform, we conducted rigorous preprocessing steps on the Sentinel-2 L2A images, including atmospheric correction, cloud removal, and pixel value normalization, to ensure high data quality and reduce the influences of the atmosphere and clouds [40]. We focused on specific bands, including B2 (blue), B3 (green), B4 (red), B5 to B8 (red edge), B8A (red edge), B11 (SWIR 1), and B12 (SWIR 2). For bands not originally at a 10-m resolution, we applied bilinear interpolation-based resampling to unify them to a 10-m resolution, generating high-precision multispectral RSIs for the study areas. The imaging information for each region is detailed in Table 1.

By combining high-resolution Google Earth imagery with visual interpretations from remote sensing experts, we meticulously annotated the FRA-covered and background areas to generate labeled sample images of the remote sensing data. We subsequently performed sliding window cropping on the sample and label images acquired from each region using a step size of 256 pixels, resulting in 2518 pairs of 512 × 512 images and labels. During this process, we excluded purely background areas without FRA but retained mixed areas with FRA-adjacent land or tidal flats as negative samples to aid the model training process, aiming to reduce the false detection rates and enhance the robustness of the model. To further improve the generalizability of the model, we applied data augmentation techniques, including horizontal flipping, vertical flipping, and diagonal mirroring, expanding the CHN-YE7-FRA dataset to 10,072 pairs of image and label blocks. The dataset was randomly divided into training, test, and validation sets at a 7:2:1 ratio, resulting in 7051 image pairs for training, 2014 images for testing, and 1007 images for validation.

## 3. Methodology

### 3.1. Overall MSSFNet Architecture

**Figure 2:**
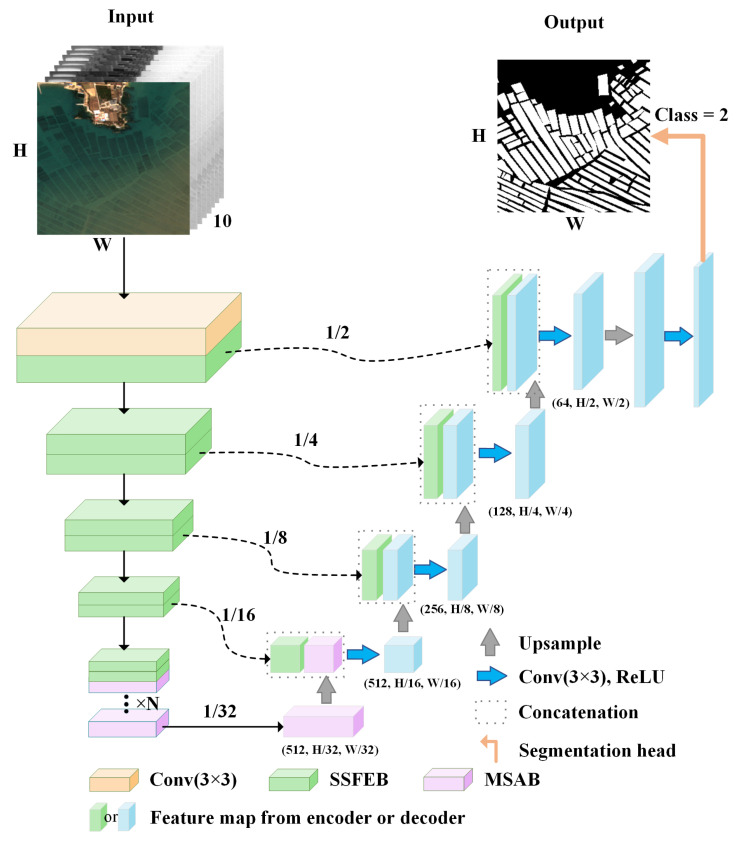
Structure of MSSFNet.

We designed MSSFNet to improve the accuracy of offshore FRA region extraction in complex marine environments. The network structure is based on the classic UNet semantic segmentation network [41], which contains two parts: an encoder and a decoder. In MSSFNet, we innovatively design a spatial–spectral feature extraction block (SSFEB), which is used to replace the ResBlock in ResNet18 [42] as the encoder of the model. The SSFEB is able to simultaneously extract multilevel spatial features and links between spectral channels, realizing the efficient fusion of spatial and spectral information. In addition, the SSFEB captures the complementary information between spectral bands and integrates it into the spatial domain, which enhances the comprehensiveness and richness of the feature representations. This design not only enhances the ability of the model to perceive FRA features in different bands but also improves the complementarity of the spectral and spatial features so that the model can accurately extract the target region even under complex backgrounds. In addition, we designed a multiscale spatial attention block (MSAB) to capture spatial features at different scales. The MSAB is able to integrate spatial information acquired under different receptive fields to realize a global receptive field and multiscale learning, which improves the ability of the model to detect various FRA features. By integrating multiscale features, the MSAB enhances the ability of the network to detect FRA regions of different sizes and shapes and improves its adaptability to complex backgrounds. Through the synergy of the SSFEB and MSAB, MSSFNet effectively integrates and utilizes multiscale spatial and spectral features, which significantly improves the extraction accuracy achieved for FRA regions in multispectral RSIs. This design not only optimizes the feature extraction process but also enhances the robustness and flexibility of the model in addressing complex marine environments so that it can better handle variable ocean conditions and background interference and ultimately achieve high-precision FRA region extraction. The network structure is shown in Figure 2.

### 3.2. Spatial–Spectral Feature Extraction Block

**Figure 3:**
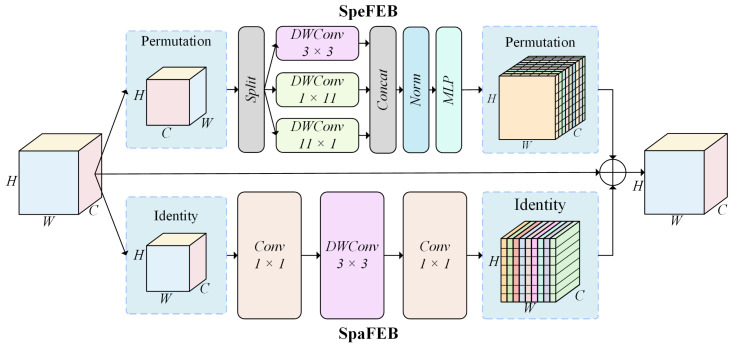
Structure of the SSFEB.

A multispectral RSI not only contains the spatial information of the feature surface but also enriches its unique spectral feature information [43]. However, the traditional convolution operation focuses on spatial features, often ignoring the interconnections between different spectral channels and failing to fully utilize the complementarity between spatial and spectral features [44]. This limitation leads to traditional methods failing to fully exploit the rich information contained in images when processing multispectral RSIs. To solve this problem, we designed the spatial–spectral feature extraction block (SSFEB) to simultaneously capture and fuse the features observed in the spatial and spectral domains. As shown in Figure 3, the SSFEB consists of a spatial feature extraction branch (SpaFEB) and a spectral feature extraction branch (SpeFEB), which significantly enhance the ability of the network to fuse information in different spectral channels and at different spatial scales through the combination of these two feature extraction mechanisms.

The SpeFEB focuses on extracting the spectral connections between spectra from the individual spectral channels of a multispectral image. This branch fully exploits the spectral differences and complementarities among the channels through individual convolutional operations conducted for each spectral channel. This approach enables the network to perceive and utilize subtle spectral information changes to effectively discriminate the features of the target objects in different bands. In this extraction branch, the dimensions of the input feature map are permuted from $X\in R^{H\times W\times C}$ to $X\in R^{H\times C\times W}$ to facilitate spectral domain processing, and the map is divided into three sub-feature maps along the W dimension, each of which applies a depthwise separable convolution (DWConv) of a different size to determine the interactions between different spectral pixels. It includes DWConv with a convolution kernel size of 3×3 for adjacent spectral feature extraction and two DWConvs with a convolution kernel size of 1×11 for horizontal and vertical long-distance spectral correlation extraction. The contextual links between different channels can be efficiently constructed through the extraction of local and long-range spectral features. After the spectral information acquired at different scales is fused, it is processed via normalization (Norm) and a multilayer perceptron (MLP) to enhance the nonlinear representations of the spectral features. The final feature map is rearranged into the original dimensions $K_{\mathrm{spe}}\in R^{H\times W\times C}$ and fused with the original input features and the output of the spatial feature extraction branch. The expression for doing so is as follows:

(1) $$x_{1},x_{2},x_{3}=\psi \left(x^{P}\right),$$

(2) $$F\left(x\right)=C({\mathrm{DW}}_{3\times 3}\left(x_{1}\right),{\mathrm{DW}}_{1\times k}\left(x_{2}\right),{\mathrm{DW}}_{k\times 1}\left(x_{3}\right)),$$

(3) $$K_{\mathrm{spe}}\;\left(x\right)=MLP{\left(\left(Norm\left(F\left(x\right)\right)\right)\right)}^{P},$$

where $x^{P}$ denotes the permutation of the input features $X\in R^{H\times W\times C}$ into $X\in R^{H\times C\times W}$. ψ denotes a splitting operation implemented on the input features. ${\mathrm{DW}}_{n\times m}$ denotes a depth-separable convolution operation with a convolution kernel size of n×m, and C denotes a concatenation operation executed along the channel dimensions. Norm denotes a BatchNorm operation, and MLP signifies multilayer perceptron processing.

The SpaFEB focuses on extracting spatial features from the input image, and it uses a series of convolutional operations to efficiently capture the local and global spatial features of the image. These operations are able to resolve the spatial distributions and morphologies of different features in detail, providing detailed spatial information for the network. In this extraction branch, point-by-point convolution is first applied to project $X\in R^{H\times W\times C}$ along the *C* dimension at low levels to reduce the subsequent computational cost. Spatial feature extraction is subsequently performed via DWConv to extract features efficiently while retaining relatively few parameters. Finally, point-by-point convolution is used to map the number of channels back to the original input to help preserve and enhance the expressions of the features, generating $K_{\mathrm{spa}}\in R^{H\times W\times C}$ spatial features, which are expressed as follows:

(4) $$K_{\mathrm{spa}}\left(x\right)=Conv_{1\times 1}\left(DW_{3\times 3}\left(Conv_{1\times 1}\left(x\right)\right)\right).$$

Finally, to effectively fuse the spectral information $K_{\mathrm{spe}}$ and the spatial information $K_{\mathrm{spa}}$, the information generated from the above two parts is fused with the original input information via element-by-element summation to establish a joint spatial–spectral feature $F_{G}\in R^{H\times W\times C}$, which enhances the ability of the network to extract and express the complex features contained in the multispectral image; this step is expressed as follows:

(5) $$F_{G}\left(x\right)=K_{\mathrm{spa}}\left(x\right)+K_{\mathrm{spe}}\left(x\right)+x.$$

In the SSFEB, these two branches integrate the extracted spatial and spectral features through a feature fusion mechanism. The fused features not only retain the structural information in the spatial domain but also incorporate the rich band information derived from the spectral domain. This combination enables the SSFEB to capture both the spatial and spectral details of the input image, which substantially improves the ability of the model to recognize target regions in multispectral RSIs.

### 3.3. Multiscale Spatial Attention Block

**Figure 4:**
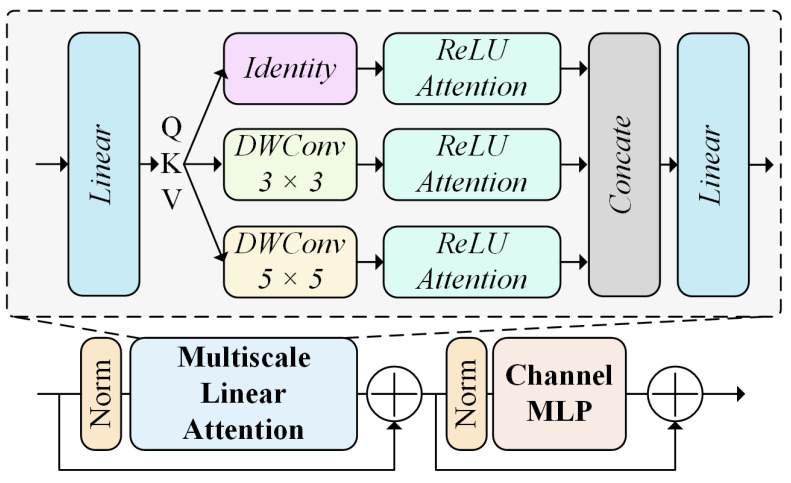
Structure of the MSAB.

RSIs cover wide geographical areas, so the target features appear at different scales and locations in the images. However, the semantic features of similar targets remain highly correlated at different spatial locations or at different scales, remaining independent of location changes or size differences [45]. Therefore, capturing multiscale spatial features and global contextual information from an RSI is essential for accurately distinguishing different FRA area sizes from the background. To address this challenge, we designed the multiscale spatial attention block (MSAB). The MSAB enhances the recognition and background differentiation effects achieved for the feature targets in complex RSIs by combining multiscale convolution and lightweight linear attention mechanisms. This block uses DWConvs of different sizes to capture multiscale spatial features and achieves effective global contextual information modeling through rectified linear unit (ReLU) attention [46]. This design enables the MSAB to adaptively assign feature weights at different locations to capture multiscale contextual information, thus accurately distinguishing between feature targets with different sizes and morphologies and improving the accuracy and reliability of remote sensing image analyses. The structure of the MSAB is shown in Figure 4.

The MSAB first linearly transforms the input feature map *X* to generate three feature mappings: a query (*Q*), a key (*K*), and a value (*V*). Then, *Q*, *K*, and *V* are subjected to multiscale aggregation, and their multiscale spatial information is extracted via DWConv operations of different sizes. The multiscale aggregated *Q*, *K*, and *V* are subsequently processed with ReLU attention, which activates *Q* and *K* and computes the output via linear attention. The output of the linear attention mechanism is finally projected through a 1×1 convolution to integrate the multiscale results, and the residuals are summed with the original inputs. Finally, the output is processed by Norm and an MLP to increase the nonlinear expressiveness of the multiscale spatial features. The specific representations are as follows:

(6) QKV=Linear(X),

(7) $$QKV_{mutilscale}=\left\{DW_{Scale}\left(QKV\right)|scale\in scales\right\},$$

(8) $$QKV\in R^{B\times C\times H\times W}\to QKV\in R^{B\times (H\;\cdot \;W)\times C},$$

(9) Q,K,V=split(QKV),

(10) $$Q^{\prime }=\mathrm{ReLU}\left(Q\right),K^{\prime }=\mathrm{ReLU}\left(K\right),$$

(11) $$A=Q^{\prime }\;\cdot \;\left(K^{\prime T}\;\cdot \;V\right),$$

(12) O=Linear(A)+X,

(13) MLP=Linear(σ(Linear(O))),

(14) $$F_{mutilscale}=MLP+O,$$

where Linear is the convolution kernel size of the 1×1 linear mapping operation. DW is the depthwise separable convolution operation with a convolution kernel size of scale×scale, where scales are 3 and 5. split is the splitting operation implemented along the *C* dimension. σ is the Gaussian error linear unit (GeLU) activation function.

### 3.4. Implementation Details

To verify the feasibility and superior performance of MSSFNet in FRA extraction tasks and ensure the fairness of the subsequent experiments, the hyperparameters of the comparison models are the same as those of MSSFNet, and all the models are trained and tested under the same hardware and software conditions. This experiment is conducted on a server equipped with 10 GB RTX 3080 GPUs, and the experimental model is implemented via the PyTorch 2.0.2 framework, with adaptive moment estimation (Adam) [47] as the optimizer, a batch size of 8, equally weighted binary cross-entropy (BCE) [48] and Dice coefficient [49] losses from the loss function, and cosine annealing [50] as the learning rate decay strategy, the initial learning rate is 0.0001. To improve the generalizability of the networks, random data augmentation processes, including random scaling, warping, flipping, and contrast transformations, are used during network training. Each network is trained for 50 epochs under the above configuration, and the resulting accuracy is verified.

### 3.5. Evaluation Metrics

In this work, the actual FRA distribution of the validation dataset is used as the benchmark for quantitative evaluation purposes, the extraction accuracies of different algorithms are compared, and reasonable evaluations and analyses are performed. The main evaluation indicators used are the F1 score (F1), intersection over union (IoU), and kappa coefficient (Kappa) metrics. The calculation formulas of the above evaluation indicators are as follows:

(15) $$F1=2\;\cdot \;\frac{\mathrm{Pr}\;\cdot \;\mathrm{Rec}}{\mathrm{Pr}+\mathrm{Rec}},$$

(16) $$IoU=\frac{TP}{FN+FP+TP},$$

(17) $$Kappa=\frac{OA-EA}{1-EA},$$

where TP is the number of true positives, TN is the number of true negatives, FP is the number of false positives, and FN is the number of false negatives. Pr, Rec, OA, and EA are defined as follows:

(18) $$Pr=\frac{TP}{TP+FP},$$

(19) $$Rec=\frac{TP}{TP+FN},$$

(20) $$OA=\frac{TP+TN}{TP+TN+FP+FN},$$

(21) $$EA=\frac{\left(TP+FN)(TP+FP)+(TN+FN)(TN+FP\right)}{{\left(TP+TN+FP+FN\right)}^{2}}.$$

## 4. Results

### 4.1. Comparative Experiments

To comprehensively and quantitatively evaluate the performance of the proposed method, seven advanced and commonly used models for semantically segmenting RSIs are selected for experimental comparisons in this paper. These models include UNet [41], UNet++ [51], DeepLabv3+ [52], and HRNet [53], which are based on CNNs, and SwinUNet [54], SegFormer [55], TCNet [56], and UNetFormer [57], which are based on ViTs. The quantitative comparison results yielded by the different methods are shown in Table 2, and the utilized evaluation metrics include the F1 score, IoU, and kappa coefficient.

On the CHN-YE7-FRA dataset constructed in this paper, our proposed MSSFNet approach achieves excellent performance, with F1 score, IoU, and kappa coefficient values of 90.61%, 82.84%, and 89.59%, respectively, outperforming the other compared methods. These results fully demonstrate the effectiveness of MSSFNet in terms of optimizing the feature extraction process, improving model robustness, and achieving high-precision FRA region extraction. In the comparison experiments, UNet, a CNN-based model, achieves the best performance, with an F1 score of 87.89%, an IoU of 78.39%, and a kappa coefficient of 86.56%. In contrast, MSSFNet improves these metrics by 2.87%, 4.69%, and 3.19%, respectively. TCNet, the top-performing ViT-based model, achieves an F1 score of 88.88%, an IoU of 79.98%, and a kappa coefficient of 87.67%. Compared with TCNet, MSSFNet achieves enhancements of 1.73%, 2.86%, and 1.92% with respect to these metrics. These improvements are attributed mainly to the SSFEB in MSSFNet, which effectively integrates spatial and spectral features, enabling the model to more accurately capture the details and boundaries of target areas. Additionally, the MSAB enhances the ability of the model to perceive spatial features at different scales, allowing for more precisely extracted and utilized FRA regions of various sizes and shapes. In summary, MSSFNet, with its innovative network structure design, excels in the complex task of FRA extraction in marine environments, significantly increasing the output F1 score, IoU, and kappa coefficient and, thus, validating its potential in remote sensing image-based FRA extraction tasks.

**Figure 5:**
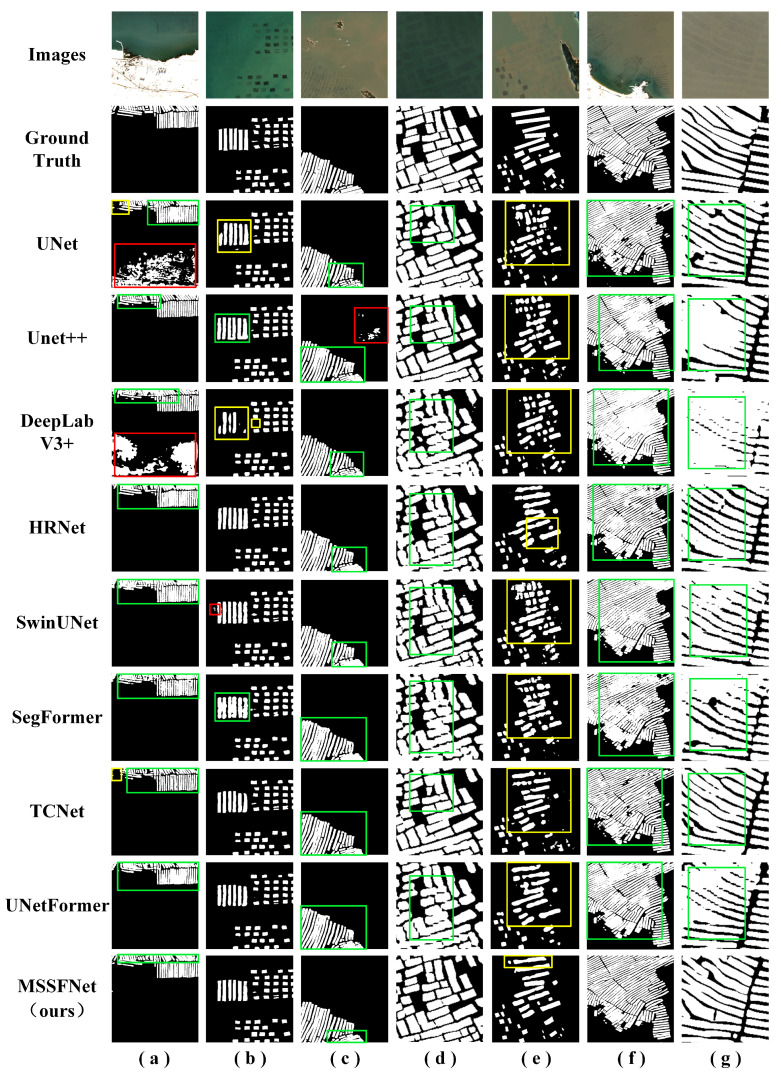
Visual comparison between MSSFNet and other mainstream networks (Subfigures (**a**–**g**) represent various scenarios. The white area in the prediction result map indicates the FRA and the black area indicates the background area. The red rectangle indicates the false detection region, the yellow rectangle indicates the missed detection region, and the green rectangle indicates the overlapping region).

To further illustrate the differences among the evaluation indices, several representative areas are selected to visually analyze the classification results produced by the various methods. In Figure 5, the first two rows correspond to the real environment and the corresponding ground-truth labels. The next few lines show the extraction results produced by different models.

The visualization results indicate that MSSFNet has significant advantages over the other eight comparison networks in offshore FRA extraction tasks. In scenarios (a), (b), and (e), the innovative design of MSSFNet effectively integrates spatial features and spectral information at different levels, significantly reducing the number of false detections and missed detections output by the model. Especially in complex marine environments with variable backgrounds, MSSFNet exhibits outstanding adaptability, effectively handles background interference, and ensures that the target areas are efficiently extracted. In scenarios (c), (f), and (g), MSSFNet effectively detects the boundaries of densely arranged FRA areas, significantly reducing the adhesion phenomena in the target regions and ensuring clear and accurate segmentation boundaries. In scenarios (d) and (e), the other networks display varying degrees of missed detections and adhesion phenomena, whereas MSSFNet more accurately detects FRA areas of different sizes and shapes in complex marine environments, showing stronger robustness and detection capabilities. In general, the performance achieved by MSSFNet in various complex scenes proves its outstanding FRA extraction ability, providing more accurate and reliable results. Its remarkable advantages in boundary division and multi-size target detection and its adaptability to complex backgrounds make it an effective tool for extracting FRA areas from challenging marine environments.

The quantitative and qualitative analyses of the aforementioned network models indicate that the MSSFNet model significantly outperforms the other methods. This method effectively mitigates the inaccurate edge segmentation issue encountered when extracting FRA from RSIs, resulting in smoother feature edges. Additionally, through the synergistic actions of the SSFEB and MSAB, MSSFNet effectively addresses the incomplete FRA extraction problem, substantially reducing the number of false positives and false negatives caused by the presence of similar shapes and spectral features. Therefore, the MSSFNet model excels at extracting offshore FRA regions from RSIs.

### 4.2. Ablation Study

To better understand the effectiveness of the different modules included in the proposed network for FRA extraction tasks, an ablation analysis was conducted on the CHN-YE7-FRA dataset. With a UNet model with ResNet18 as its encoder as the baseline, the SSFEB was used to replace the residual blocks in ResNet18, followed by the addition of the MSAB. This was done to illustrate the contribution of each component to the overall performance improvement achieved by the network. The experimental results are shown in Table 3.

The experimental results show that after integrating the SSFEB on top of the baseline, the model performance improved significantly. The SSFEB efficiently integrated spatial features at different hierarchical levels with spectral information, making the model more accurate in terms of distinguishing the target areas from backgrounds. Specifically, the F1 score increased by 2.40%, the IoU increased by 3.91%, and the kappa coefficient increased by 2.67%, indicating that the SSFEB increased the detection accuracy and consistency of the network. With the further addition of the MSAB, the model performance improved again. The MSAB captured spatial features at different scales, enhancing the perception capabilities of the network in multiscale FRA regions. Compared with those produced with the SSFEB alone, the F1 score, IoU, and kappa coefficient improved by 0.47%, 0.78%, and 0.52%, respectively, which reflects the fine processing ability of the MSAB in terms of extracting target features from complex areas. In conclusion, the synergistic effects of SSFEB and MSAB enable MSSFNet to extract FRA regions accurately in complex marine environments, significantly improving its segmentation accuracy and robustness.

**Figure 6:**
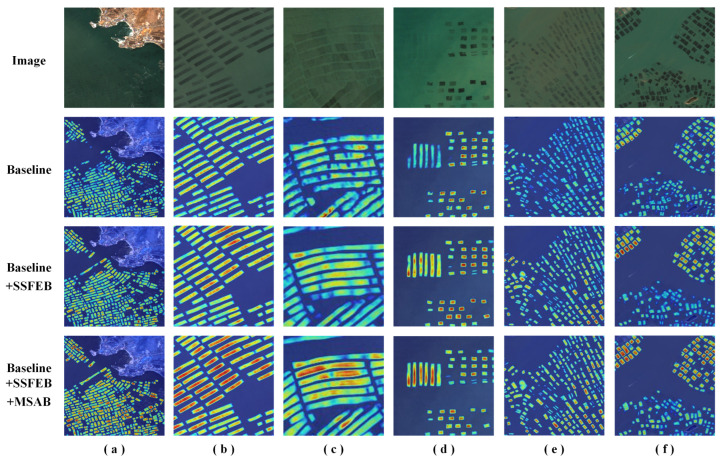
Comparison among the attention visualizations produced for the modules integrated into MSSFNet (Subfigures (**a**–**f**) represent various scenarios. The results of the extraction are plotted from blue to red to indicate low to high levels of attention, respectively).

The above conclusions demonstrate the necessity of each module in the proposed model for obtaining optimal FRA extraction results. To further illustrate the role of each module, we conducted an attention visualization analysis via gradient-weighted class activation mapping (Grad-CAM) [58] on different modules, aiming to increase the interpretability of the modules used in this study. The results are shown in Figure 6.

The attention visualization results indicate that the baseline model, while capable of recognizing and extracting FRA regions, exhibited several shortcomings. For example, it tended to produce blurry details and adhesive phenomena in edge regions, and it missed small targets during the detection process. With the introduction of the SSFEB, the model significantly increased its focus on FRA regions, particularly improving its ability to identify smaller targets. Owing to the efficient integration of spatial and spectral features, the delineated object boundaries became clearer, markedly reducing edge blurriness and adhesive effects. Furthermore, introducing the MSAB enhanced the performance of the model in terms of handling details even more. The attention paid by the network to subtle FRA regions intensified, resulting in clearer and more refined boundaries for FRA areas.

Overall, the gradual addition of the SSFEB and MSAB to the baseline model not only significantly improved the quantitative indicators but also significantly improved the attention visualization results. Therefore, the designs of the SSFEB and MSAB modules are effective and robust, and they can improve the accuracy and clarity of the model with respect to extracting offshore FRA regions under complex sea conditions.

## 5. Discussion

### 5.1. Application of the Model

**Figure 7:**
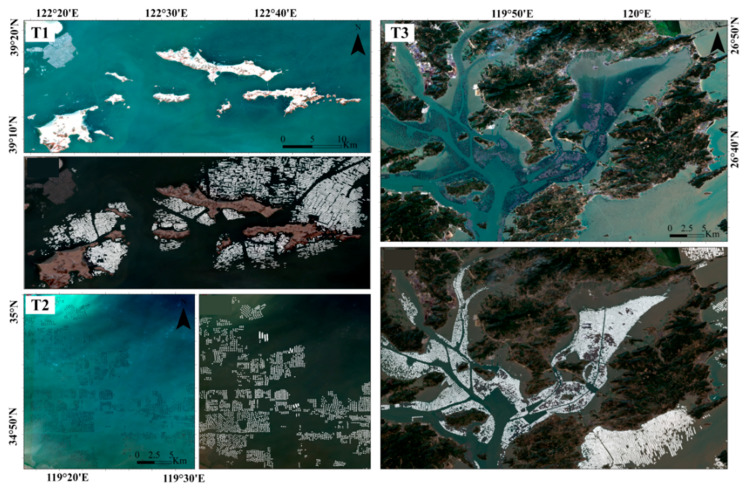
The FRA distribution areas of Changhai County, Haizhou Bay, and Sansha Bay in 2024 (the white areas in the figure represent the FRA regions, with T1 denoting Changhai County, T2 signifying Haizhou Bay, and T3 denoting Sansha Bay).

To verify the practical application capability of the model, we applied the trained MSSFNet model to extraction tasks in the FRA regions of Changhai County, Haizhou Bay, and Sansha Bay in January 2024. First, we acquired Sentinel-2 images with low cloud cover in January 2024 through the GEE platform. Using the MSSFNet model, we subsequently extracted the aquaculture areas from these three FRA regions with typical geographical environments and locations, obtaining distribution maps of the areas, as shown in Figure 7.

Compared with the original images from 2024, the extraction results produced by MSSFNet are highly consistent with the actual distribution of the offshore aquaculture areas. In coastal island aquaculture, open sea aquaculture, and bay aquaculture, MSSFNet effectively identifies the FRA regions, demonstrating its robustness and accuracy across different geographical environments and latitudes. Even under complex mixed aquaculture conditions, MSSFNet can accurately extract FRA regions, highlighting its strong environmental adaptability. For example, in nearshore areas affected by sediment and water shadows, MSSFNet still reliably extracts FRA areas, indicating the stability and robustness of its extraction results.

**Figure 8:**
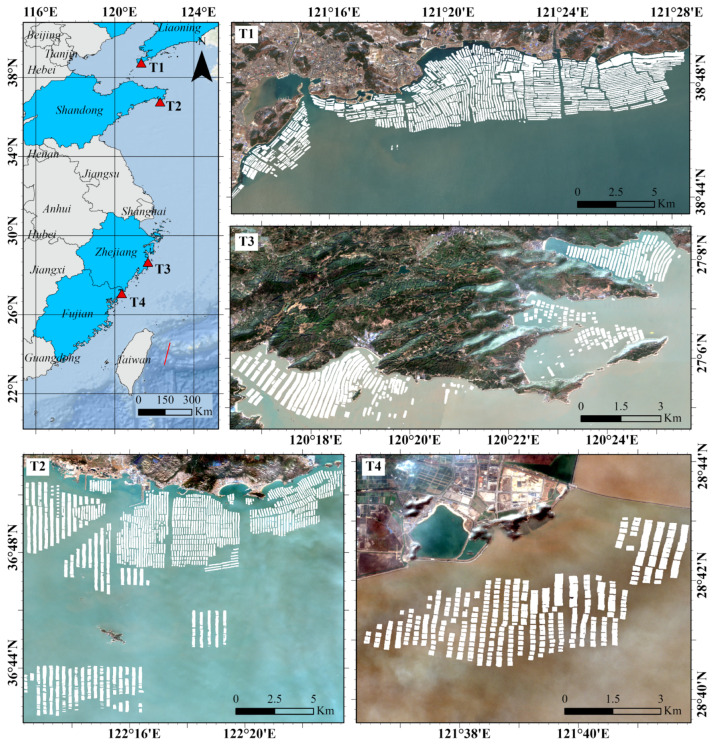
Geographical locations and FRA extraction results outside the sampling areas (T1 denotes Longwangtang Bay in Liaoning Province, T2 represents Zhangjia Bay in Shandong Province, T3 signifies Taizhou Bay in Zhejiang Province, and T4 denotes Qingchuan Bay in Fujian Province). The white areas in the figure represent the FRA extraction results.

To further validate the spatial stability of the MSSFNet model, we extended our verification work to offshore aquaculture areas located at four different latitudes: Longwangtang Bay in Liaoning Province, Zhangjia Bay in Shandong Province, Taizhou Bay in Zhejiang Province, and Qingchuan Bay in Fujian Province. Similarly, we used the GEE platform to acquire Sentinel-2 images with low cloud cover for these regions in January 2024 and successfully extracted the FRA areas. The geographical locations of these regions and the FRA extraction results produced by MSSFNet are shown in Figure 8.

The FRA extraction results outside the sampling areas indicate that, owing to the diverse coverage and rich FRA samples of the CHN-YE7-FRA dataset, combined with the powerful learning capabilities of MSSFNet, the trained MSSFNet model can comprehensively learn the complex characteristics and spatial relationships of FRA regions, thereby exhibiting excellent generalizability. Even in untrained marine areas, MSSFNet can still extract FRA regions accurately. This generalizability is crucial for FRA extraction tasks conducted in different regions. By leveraging training and transfer learning applications on the CHN-YE7-FRA dataset, MSSFNet can quickly and accurately identify and extract FRA regions across various marine areas, not only significantly enhancing the automation level of fishery resource surveys but also providing reliable technical support for the sustainable management of marine resources. This information can help decision-makers better understand and manage marine resources, thereby promoting sustainable development in fisheries.

In the future, we will apply MSSFNet for accurate monitoring of large-scale FRA to support the regulation and sustainable development of mariculture. On the one hand, we will utilize RSIs from different periods for dynamic monitoring and FRA statistics. By comparing and analyzing data from different periods, we can reveal the spatial and temporal evolution characteristics of FRA and its development trend. This information will provide efficient and accurate data support to fishery regulatory authorities, thus helping to formulate more scientific and reasonable fishery management policies. On the other hand, as an important part of the ocean carbon sink, the fishery carbon sink is a key way to achieve “carbon neutrality”, and the carbon sink capacity of FRA, as the most common and widely used offshore aquaculture method, is particularly outstanding. By quantifying FRA areas, we can assess the carbon sequestration and sink enhancement of fisheries via relevant algorithms and provide a new reference for the ecological value of the marine carbon sink capacity.

### 5.2. Advantages and Limitations

Our proposed MSSFNet demonstrates outstanding quantitative and qualitative performance on the CHN-YE7-FRA dataset in comparison with other advanced deep learning-based semantic segmentation networks. First, the introduction of the SSFEB enables MSSFNet to effectively integrate spatial and spectral information, thus capturing the details and boundaries of target areas more accurately and significantly reducing the number of false positives and missed detections produced. Additionally, the MSAB enhances the ability of the model to refine the target feature extraction process in complex regions, improving the accuracy and consistency of the output segmentation results, particularly when addressing FRA regions with different scales. Overall, MSSFNet excels at extracting FRA regions in complex marine environments. It effectively addresses background interference and significantly enhances the precision and robustness of segmentation, making it well-suited for large-scale FRA extraction applications.

Despite the excellent performance of MSSFNet in the current study, it still faces some challenges. First, multispectral RSIs capture feature information above the water surface, while there is a relative lack of semantic information for FRA regions located deeper underwater. This insufficient information may lead to the missed detection of FRA regions, especially when the underwater features are similar to the background features or when the contrast is low. Second, the variable and complex climatic conditions in the ocean have a significant effect on the quality of multispectral RSIs. For example, meteorological factors such as the build-up of cloud cover, the formation of sea spray, and changes in light can significantly affect the clarity and quality of images. The thicker cloud cover may obscure the FRA areas, resulting in the loss of some or all of the FRA feature information and, thus, affecting the accurate monitoring of FRA. These meteorological disturbances limit all-weather and full-time offshore FRA monitoring missions, which in turn affects data coherence and monitoring accuracy.

To address these challenges, in the future, we plan to incorporate prior knowledge of RSIs (e.g., the water body index, vegetation index) [59] into network training to enhance the differences between FRA areas and the background and provide richer semantic information. In addition, with advancements in remote sensing technology, the integration of multisource RSIs will become a crucial direction for future development [60]. By combining data acquired from various remote sensing sources, such as synthetic aperture radar (SAR) and microwaves, we can obtain more comprehensive semantic FRA information and reduce the influence of external environmental and climatic factors on the extraction accuracy of FRA areas, thus realizing all-weather monitoring and extraction tasks. With the help of advanced image processing techniques and data fusion methods, we expect to overcome the existing challenges and achieve more accurate and reliable FRA monitoring, which will provide stronger support for marine resource management and environmental protection.

## 6. Conclusions

In this paper, we propose a novel semantic segmentation network, MSSFNet, which is designed to extract offshore FRA regions from multispectral RSIs. Compared with the existing methods, MSSFNet introduces the SSFEB and MSAB, which significantly enhance the ability to capture and integrate spectral features and spatial information from multispectral images. The SSFEB overcomes the limitations of traditional convolutional methods in terms of processing multispectral RSIs, allowing the network to more accurately capture spectral correlations and spatial features from complex environments, thereby improving the accuracy of FRA region recognition. By enhancing the ability of the model to perceive multiscale spatial features, the MSAB effectively improves the process of detecting FRA regions of varying sizes and shapes, reduces adhesion phenomena, and enhances the clarity and consistency of the output segmentation results. To validate the performance of MSSFNet, we constructed the CHN-YE7-FRA dataset on the basis of Sentinel-2 RSIs and conducted extensive experimental evaluations. We compared MSSFNet with four CNN-based models and four ViT-based models. The experimental results demonstrated that MSSFNet achieved the best performance in offshore FRA extraction tasks, with the F1 score, IoU, and kappa coefficient reaching 90.76%, 83.08%, and 89.75%, respectively. Ablation experiments further validated the effectiveness of the SSFEB and MSAB modules in enhancing the resulting extraction accuracy. Additionally, we applied the trained MSSFNet model to real-world scenarios and successfully extracted offshore FRA regions from multiple areas across different years, demonstrating the practical application value of the proposed model. However, the experiments also revealed that the extraction results of MSSFNet were still affected by the quality of the input data and the complexity of the analyzed environment, which led to some omissions and adhesion. To solve these problems, future research will focus on incorporating prior knowledge derived from remote sensing data to further optimize the quality of the data, explore the integration of multisource remote sensing data, and collect more samples to increase the generalizability of the model. In summary, this study provides an innovative and efficient solution for the high-precision extraction of offshore FRA regions from multispectral RSIs through the design and validation of MSSFNet, demonstrating its significant potential for improving extraction accuracy and practical applications. Future efforts will be dedicated to applying MSSFNet to more extensive offshore aquaculture areas monitoring and extraction tasks, driving the application and development of remote sensing technology in fishery resource management scenarios.

## Figures and Tables

**Table 1:** Overview of the Sentinel-2 images contained in the CHN-YE7-FRA dataset.

Areas	Image Size	Geographical Scope	Coverage Area (km^2^)	Image Dates
Changhai County	4450 × 2476	122°29′ E, 39°13′ N ∼122°82′ E, 39°35′ N	110.18	1 December 2022 and 25 January 2023
Jinshitan Bay	5879 × 238	121°90′ E, 38°93′ N ∼122°30′ E, 39°15′ N	140.33	1 December 2022 and 25 January 2023
Rongcheng Bay	3181 × 5927	122°45′ E, 36°87′ N ∼122° 73′ E, 37°41′ N	188.54	1 December 2022 and 25 January 2023
Haizhou Bay	4096 × 3568	119°22′ E, 34°75′ N ∼119°59′ E, 35°7′ N	146.15	5 December 2022 and 20 January 2023
Dayu Bay	1065 × 902	120°52′ E, 27°32′ N ∼120°62′ E, 27°40′ N	9.61	20 December 2022 and 25 January 2023
Sansha Bay	6101 × 4092	119°59′ E, 26°50′ N ∼120°14′ E, 26°86′ N	249.65	21 December 2022 and 25 January 2023
Zhaoan Bay	1239 × 2121	117°25′ E, 23°55′ N ∼117°36′ E, 23°74′ N	26.28	5 December 2022 and 25 January 2023

Each geographical scope in the table consists of the latitude and longitude range of the corresponding image.

**Table 2:** Quantitative evaluation of the extraction results produced by different models.

Method	F1 (%)	IoU (%)	Kappa (%)
UNet	87.89	78.39	86.56
UNet++	87.17	77.26	85.78
DeepLabv3+	81.04	68.12	78.92
HRNet	86.95	76.92	85.54
SwinUNet	86.57	76.33	85.08
SegFormer	87.02	77.02	85.60
TCNet	88.88	79.98	87.67
UNetFormer	87.16	77.25	85.76
**MSSFNet (ours)**	**90.76**	**83.08**	**89.75**

The best performance is marked in bold.

**Table 3:** Results of ablation experiments conducted with different combinations of modules.

Name	F1 (%)	IoU (%)	Kappa (%)
Baseline	87.89	78.39	86.56
+SSFEB	90.29	82.30	89.23
+SSFEB +MSAB	**90.76**	**83.08**	**89.75**

The best performance is marked in bold.

## Data Availability

Our code and dataset are available at the following link: https://github.com/Harsh-M1/MSSFNet (accessed on 15 July 2024).

## Abbreviations

The following abbreviations are used in this manuscript:

FRA	floating raft aquaculture
GEE	Google Earth Engine
RSIs	remote sensing images
SSFEB	spatial–spectral feature extraction block
MSAB	multiscale spatial attention block
MSSFNet	multiscale spatial–spectral fusion network
CNNs	convolutional neural networks
DC	dilated convolution
ViT	Vision Transformer
